# Pyrrolo­[2,1-*c*][1,4]benzodiazepine-5,11-dithione

**DOI:** 10.1107/S1600536810021410

**Published:** 2010-06-16

**Authors:** Sarah Ourahou, Hafid Zouihri, El Mokhtar Essassi, Seik Weng Ng

**Affiliations:** a Laboratoire de Chimie Organique Hétérocyclique, Pôle de Compétences Pharmacochimie, Université Mohammed V-Agdal, BP 1014 Avenue Ibn Batout, Rabat, Morocco; b CNRST Division UATRS, Angle Allal Fassi/FAR, BP 8027 Hay Riad, Rabat, Morocco; c Department of Chemistry, University of Malaya, 50603 Kuala Lumpur, Malaysia

## Abstract

The seven-membered fused-ring in the title compound, C_12_H_12_N_2_S_2_, adopts a boat conformation (with the two phenyl­ene C atoms representing the stern and the methine C atom the prow). This methine C atom and the tertiary N atom also belong to a five-membered ring, which has an envelope conformation. In the crystal structure, mol­ecules are linked about a center of inversion by pairs of N—H⋯S hydrogen bonds.

## Related literature

For background to pyrrolo­[2,1-*c*][1,4]benzodiazepine-5,11-dione, see: Antonow *et al.* (2007 ▶); Kamal *et al.* (2007 ▶). For a related structure, Neidle *et al.* (1991 ▶).
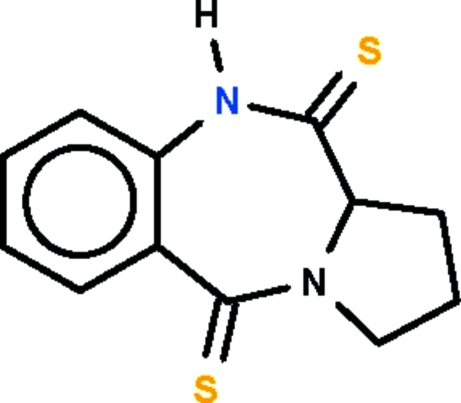


## Experimental

### 

#### Crystal data

C_12_H_12_N_2_S_2_
*M* *_r_* = 248.36
Monoclinic, 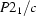

*a* = 13.9831 (5) Å
*b* = 10.0134 (3) Å
*c* = 8.2670 (3) Å
β = 97.089 (1)°
*V* = 1148.68 (7) Å^3^
*Z* = 4
Mo *K*α radiation
μ = 0.44 mm^−1^
*T* = 200 K
0.12 × 0.10 × 0.07 mm

#### Data collection

Bruker X8 APEXII diffractometer
Absorption correction: multi-scan (*SADABS*; Sheldrick, 1996 ▶) *T*_min_ = 0.950, *T*_max_ = 0.970
14013 measured reflections
3017 independent reflections
2117 reflections with *I* > 2σ(*I*)
*R*_int_ = 0.055

#### Refinement

*R*[*F* ^2^ > 2σ(*F* ^2^)] = 0.042
*wR*(*F* ^2^) = 0.107
*S* = 1.01
3017 reflections
193 parameters
12 restraints
All H-atom parameters refined
Δρ_max_ = 0.39 e Å^−3^
Δρ_min_ = −0.28 e Å^−3^

### 

Data collection: *APEX2* (Bruker, 2008 ▶); cell refinement: *SAINT* (Bruker, 2008 ▶); data reduction: *SAINT*; program(s) used to solve structure: *SHELXS97* (Sheldrick, 2008 ▶); program(s) used to refine structure: *SHELXL97* (Sheldrick, 2008 ▶); molecular graphics: *X-SEED* (Barbour, 2001 ▶); software used to prepare material for publication: *publCIF* (Westrip, 2010 ▶).

## Supplementary Material

Crystal structure: contains datablocks global, I. DOI: 10.1107/S1600536810021410/xu2774sup1.cif

Structure factors: contains datablocks I. DOI: 10.1107/S1600536810021410/xu2774Isup2.hkl

Additional supplementary materials:  crystallographic information; 3D view; checkCIF report

## Figures and Tables

**Table 1:** Hydrogen-bond geometry (Å, °)

*D*—H⋯*A*	*D*—H	H⋯*A*	*D*⋯*A*	*D*—H⋯*A*
N1—H1⋯S1^i^	0.86 (1)	2.58 (1)	3.411 (2)	166 (2)

Symmetry code: (i) 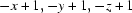.

**Fig. 1.**
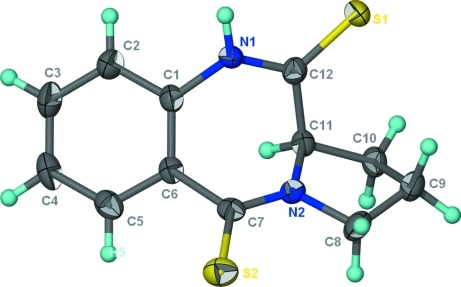
Thermal ellipsoid plot (Barbour, 2001) of the molecule of C12H12N2S2 at the 70% probability level; hydrogen atoms are drawn as spheres of arbitrary radius.

## supplementary crystallographic information

### Comment

Pyrrolo[2,1-*c*][1,4]benzodiazepine-5,11-dione is the homolog of a class of compounds that are active against mycobacterium tuberculosis (Kamal *et al.*, 2007). Other C-2 aryl substituted derivatives are cytotoxic (Antonow *et al.*, 2007). The crystal structure of the parent compound has not been reported although that the (11*aS*)-1,2,3,10,11,11*a*-hexahydro has bee published (Neidle *et al.*, 1997). The structure of the parent compound is probably similar to that of the isoelectronic dithione (Scheme I, Fig. 1). The seven-membered fused-ring in C_12_H_12_N_2_S_2_ adopts a boat conformation (with the two phenylene carbons representing the stern and the methine carbon atom the prow). This methine C atom and the tertiary N atom also belong to a five-membered ring, which has an envelope shape. Two C_12_H_12_N_2_O_2_ molecules are linked about a center-of-inversion by *N*–*H*···O_carbonyl_ hydrogen bonds.

### Experimental

Pyrrolo[2,1-*c*][1,4]benzodiazepine-5,11-dithione (1 g, 4.62 mmol) and phosphorus pentasulfide (2.05 g, 9.24 mmol) are heated in pyridine (60 ml) for 4 h. The pyridine was evaporated under reduced pressure and the residue heated in water (100 ml). The suspension was set aside for a day. The insoluble product was recrystallized from ethanol to furnish colorless crystals (90% yield).

### Refinement

The nitrogen- and carbon-bound H-atoms were refined with restraints (C–H 0.95±0.01 Å for the aromatic atoms and 0.99±0.01 Å for the aliphatic atoms; N–H 0.86±0.01 Å). Their temperature factors were freely refined.

### Crystal data

**Table:**

C_12_H_12_N_2_S_2_	*F*(000) = 520
*M**_r_* = 248.36	*D*_x_ = 1.436 Mg m^−^^3^
Monoclinic, *P*2_1_/*c*	Mo *K*α radiation, λ = 0.71073 Å
Hall symbol: -P 2ybc	Cell parameters from 2753 reflections
*a* = 13.9831 (5) Å	θ = 2.5–26.5°
*b* = 10.0134 (3) Å	µ = 0.44 mm^−^^1^
*c* = 8.2670 (3) Å	*T* = 200 K
β = 97.089 (1)°	Prism, colorless
*V* = 1148.68 (7) Å^3^	0.12 × 0.10 × 0.07 mm
*Z* = 4	

### Data collection

**Table:**

Bruker X8 APEXII diffractometer	3017 independent reflections
Radiation source: fine-focus sealed tube	2117 reflections with *I* > 2σ(*I*)
graphite	*R*_int_ = 0.055
φ and ω scans	θ_max_ = 28.9°, θ_min_ = 1.5°
Absorption correction: multi-scan (*SADABS*; Sheldrick, 1996)	*h* = −18→18
*T*_min_ = 0.950, *T*_max_ = 0.970	*k* = −12→13
14013 measured reflections	*l* = −11→11

### Refinement

**Table:**

Refinement on *F*^2^	Primary atom site location: structure-invariant direct methods
Least-squares matrix: full	Secondary atom site location: difference Fourier map
*R*[*F*^2^ > 2σ(*F*^2^)] = 0.042	Hydrogen site location: inferred from neighbouring sites
*wR*(*F*^2^) = 0.107	All H-atom parameters refined
*S* = 1.01	*w* = 1/[σ^2^(*F*_o_^2^) + (0.047*P*)^2^ + 0.5062*P*] where *P* = (*F*_o_^2^ + 2*F*_c_^2^)/3
3017 reflections	(Δ/σ)_max_ = 0.001
193 parameters	Δρ_max_ = 0.39 e Å^−^^3^
12 restraints	Δρ_min_ = −0.28 e Å^−^^3^

### Fractional atomic coordinates and isotropic or equivalent isotropic displacement parameters (Å^2^)

**Table:**

	*x*	*y*	*z*	*U*_iso_*/*U*_eq_	
S1	0.60817 (4)	0.35830 (6)	0.60020 (8)	0.02972 (16)	
S2	0.92223 (4)	0.75918 (6)	0.51650 (7)	0.02991 (16)	
N1	0.62240 (12)	0.61932 (18)	0.5884 (2)	0.0221 (4)	
N2	0.82707 (11)	0.57256 (17)	0.6540 (2)	0.0196 (4)	
C1	0.65476 (14)	0.7511 (2)	0.6273 (2)	0.0205 (4)	
C2	0.58339 (16)	0.8462 (2)	0.6410 (3)	0.0277 (5)	
C3	0.60718 (17)	0.9769 (2)	0.6798 (3)	0.0318 (5)	
C4	0.70347 (18)	1.0143 (2)	0.7079 (3)	0.0307 (5)	
C5	0.77396 (16)	0.9220 (2)	0.6894 (3)	0.0251 (5)	
C6	0.75250 (14)	0.7891 (2)	0.6467 (2)	0.0191 (4)	
C7	0.83258 (14)	0.7001 (2)	0.6109 (2)	0.0194 (4)	
C8	0.90083 (16)	0.4717 (2)	0.6291 (3)	0.0274 (5)	
C9	0.86119 (16)	0.3432 (2)	0.6929 (3)	0.0284 (5)	
C10	0.80322 (16)	0.3926 (2)	0.8260 (3)	0.0251 (5)	
C11	0.75591 (14)	0.5204 (2)	0.7555 (2)	0.0190 (4)	
C12	0.66077 (14)	0.5040 (2)	0.6465 (2)	0.0205 (4)	
H1	0.5667 (10)	0.611 (2)	0.534 (2)	0.027 (6)*	
H2	0.5176 (8)	0.822 (2)	0.628 (3)	0.034 (7)*	
H3	0.5592 (13)	1.0406 (19)	0.694 (3)	0.030 (6)*	
H4	0.7233 (16)	1.1027 (13)	0.736 (3)	0.032 (7)*	
H5	0.8403 (8)	0.948 (2)	0.708 (3)	0.021 (6)*	
H81	0.9608 (11)	0.498 (2)	0.697 (2)	0.030 (6)*	
H82	0.9116 (16)	0.471 (2)	0.5138 (14)	0.034 (7)*	
H91	0.8196 (14)	0.296 (2)	0.606 (2)	0.029 (6)*	
H92	0.9130 (13)	0.281 (2)	0.736 (3)	0.037 (7)*	
H11	0.7457 (14)	0.5865 (16)	0.8397 (19)	0.017 (5)*	
H101	0.8467 (13)	0.421 (2)	0.9245 (19)	0.029 (6)*	
H102	0.7565 (13)	0.3271 (18)	0.859 (2)	0.025 (6)*	

### Atomic displacement parameters (Å^2^)

**Table:**

	*U*^11^	*U*^22^	*U*^33^	*U*^12^	*U*^13^	*U*^23^
S1	0.0233 (3)	0.0180 (3)	0.0459 (3)	−0.0022 (2)	−0.0034 (2)	0.0011 (3)
S2	0.0248 (3)	0.0352 (4)	0.0300 (3)	−0.0034 (2)	0.0043 (2)	0.0035 (2)
N1	0.0172 (9)	0.0175 (10)	0.0299 (9)	−0.0008 (7)	−0.0039 (7)	−0.0007 (7)
N2	0.0172 (8)	0.0202 (10)	0.0211 (8)	0.0010 (7)	0.0012 (7)	−0.0007 (7)
C1	0.0238 (10)	0.0169 (11)	0.0202 (9)	0.0007 (8)	0.0004 (8)	−0.0004 (8)
C2	0.0244 (11)	0.0242 (12)	0.0344 (12)	0.0036 (9)	0.0037 (9)	−0.0019 (10)
C3	0.0360 (13)	0.0219 (13)	0.0382 (13)	0.0085 (10)	0.0074 (10)	−0.0027 (10)
C4	0.0455 (14)	0.0177 (12)	0.0289 (11)	−0.0002 (10)	0.0041 (10)	−0.0019 (9)
C5	0.0300 (12)	0.0222 (12)	0.0220 (10)	−0.0046 (9)	−0.0008 (9)	0.0023 (9)
C6	0.0232 (10)	0.0180 (11)	0.0157 (9)	0.0001 (8)	0.0013 (8)	0.0009 (8)
C7	0.0191 (10)	0.0229 (11)	0.0150 (9)	−0.0025 (8)	−0.0027 (7)	−0.0005 (8)
C8	0.0224 (11)	0.0276 (13)	0.0317 (12)	0.0081 (10)	0.0015 (9)	−0.0012 (10)
C9	0.0259 (11)	0.0223 (12)	0.0351 (12)	0.0073 (9)	−0.0042 (10)	−0.0028 (10)
C10	0.0296 (12)	0.0187 (11)	0.0254 (10)	0.0021 (9)	−0.0035 (9)	0.0012 (9)
C11	0.0221 (10)	0.0162 (10)	0.0184 (9)	0.0004 (8)	0.0016 (8)	0.0000 (8)
C12	0.0203 (10)	0.0197 (11)	0.0224 (10)	0.0019 (8)	0.0065 (8)	−0.0010 (8)

### Geometric parameters (Å, °)

**Table:**

S1—C12	1.657 (2)	C4—H4	0.947 (10)
S2—C7	1.665 (2)	C5—C6	1.400 (3)
N1—C12	1.337 (3)	C5—H5	0.956 (9)
N1—C1	1.419 (3)	C6—C7	1.489 (3)
N1—H1	0.855 (10)	C8—C9	1.520 (3)
N2—C7	1.330 (3)	C8—H81	0.984 (10)
N2—C11	1.474 (2)	C8—H82	0.984 (9)
N2—C8	1.476 (3)	C9—C10	1.527 (3)
C1—C2	1.394 (3)	C9—H91	0.986 (10)
C1—C6	1.409 (3)	C9—H92	0.986 (10)
C2—C3	1.379 (3)	C10—C11	1.523 (3)
C2—H2	0.944 (10)	C10—H101	0.996 (10)
C3—C4	1.389 (3)	C10—H102	0.986 (9)
C3—H3	0.942 (10)	C11—C12	1.521 (3)
C4—C5	1.374 (3)	C11—H11	0.984 (9)
			
C12—N1—C1	128.23 (17)	N2—C8—C9	103.87 (17)
C12—N1—H1	113.8 (16)	N2—C8—H81	107.5 (14)
C1—N1—H1	117.1 (16)	C9—C8—H81	110.5 (14)
C7—N2—C11	123.94 (16)	N2—C8—H82	109.3 (14)
C7—N2—C8	123.70 (17)	C9—C8—H82	116.0 (15)
C11—N2—C8	111.65 (16)	H81—C8—H82	109.2 (19)
C2—C1—C6	120.0 (2)	C8—C9—C10	102.99 (18)
C2—C1—N1	116.23 (18)	C8—C9—H91	110.8 (14)
C6—C1—N1	123.69 (18)	C10—C9—H91	111.3 (13)
C3—C2—C1	120.8 (2)	C8—C9—H92	112.0 (14)
C3—C2—H2	118.2 (16)	C10—C9—H92	112.0 (14)
C1—C2—H2	120.9 (16)	H91—C9—H92	108 (2)
C2—C3—C4	119.7 (2)	C11—C10—C9	103.89 (17)
C2—C3—H3	121.1 (15)	C11—C10—H101	105.2 (14)
C4—C3—H3	119.1 (15)	C9—C10—H101	111.0 (13)
C5—C4—C3	119.6 (2)	C11—C10—H102	113.1 (13)
C5—C4—H4	117.6 (15)	C9—C10—H102	114.2 (13)
C3—C4—H4	122.8 (15)	H101—C10—H102	109.0 (18)
C4—C5—C6	122.2 (2)	N2—C11—C12	107.68 (15)
C4—C5—H5	119.9 (14)	N2—C11—C10	102.94 (16)
C6—C5—H5	117.9 (14)	C12—C11—C10	116.32 (17)
C5—C6—C1	117.39 (19)	N2—C11—H11	109.3 (12)
C5—C6—C7	118.44 (18)	C12—C11—H11	107.4 (12)
C1—C6—C7	123.96 (19)	C10—C11—H11	112.9 (12)
N2—C7—C6	116.84 (17)	N1—C12—C11	113.76 (17)
N2—C7—S2	122.59 (16)	N1—C12—S1	122.01 (15)
C6—C7—S2	120.55 (16)	C11—C12—S1	124.22 (15)
			
C12—N1—C1—C2	−140.9 (2)	C5—C6—C7—S2	−36.1 (2)
C12—N1—C1—C6	41.2 (3)	C1—C6—C7—S2	138.49 (17)
C6—C1—C2—C3	−2.6 (3)	C7—N2—C8—C9	−178.51 (18)
N1—C1—C2—C3	179.4 (2)	C11—N2—C8—C9	10.8 (2)
C1—C2—C3—C4	−0.9 (3)	N2—C8—C9—C10	−30.2 (2)
C2—C3—C4—C5	3.0 (3)	C8—C9—C10—C11	38.8 (2)
C3—C4—C5—C6	−1.6 (3)	C7—N2—C11—C12	79.1 (2)
C4—C5—C6—C1	−1.8 (3)	C8—N2—C11—C12	−110.28 (18)
C4—C5—C6—C7	173.16 (19)	C7—N2—C11—C10	−157.51 (18)
C2—C1—C6—C5	3.8 (3)	C8—N2—C11—C10	13.1 (2)
N1—C1—C6—C5	−178.33 (18)	C9—C10—C11—N2	−31.7 (2)
C2—C1—C6—C7	−170.78 (19)	C9—C10—C11—C12	85.7 (2)
N1—C1—C6—C7	7.0 (3)	C1—N1—C12—C11	−6.1 (3)
C11—N2—C7—C6	−9.7 (3)	C1—N1—C12—S1	174.50 (16)
C8—N2—C7—C6	−179.26 (17)	N2—C11—C12—N1	−65.3 (2)
C11—N2—C7—S2	171.99 (14)	C10—C11—C12—N1	179.85 (18)
C8—N2—C7—S2	2.5 (3)	N2—C11—C12—S1	114.07 (17)
C5—C6—C7—N2	145.61 (19)	C10—C11—C12—S1	−0.8 (3)
C1—C6—C7—N2	−39.8 (3)		

### Hydrogen-bond geometry (Å, °)

**Table:**

*D*—H···*A*	*D*—H	H···*A*	*D*···*A*	*D*—H···*A*
N1—H1···S1^i^	0.86 (1)	2.58 (1)	3.411 (2)	166 (2)

Symmetry codes: (i) −*x*+1, −*y*+1, −*z*+1.
